# Sublingual immunotherapy tablet for the treatment of house dust mite allergic rhinitis in Canada: an alternative to minimize treatment costs?

**DOI:** 10.1186/s13223-019-0344-3

**Published:** 2019-04-27

**Authors:** Anne K. Ellis, Rémi Gagnon, Eva Hammerby, Andrea Lau

**Affiliations:** 1 Division of Allergy and Immunology, Department of Medicine, Queen’s University, Kingston, ON Canada; 2 Service d’Allergie et Immunologie, Département de médecine, CHU de Québec, Quebec, Canada; 3 ALK-Abello A/S, Hørsholm, Denmark; 4 PDCI Market Access Inc., Ottawa, ON Canada

**Keywords:** Cost-minimization, House dust mite, Sublingual immunotherapy, Allergy immunotherapy, Allergic rhinitis

## Abstract

**Background:**

A cost-minimization analysis (CMA) was performed to estimate the economic impact of introducing the SQ house dust mite sublingual immunotherapy (SQ HDM SLIT)-tablet marketed as ACARIZAX™ (regulatory approval May 2017) for the treatment of HDM-induced allergic rhinitis in Canada (Ontario and Quebec), where house dust mite subcutaneous immunotherapy (HDM SCIT) is already an available treatment option.

**Methods:**

A CMA was deemed appropriate and was based on the assumption that the SQ HDM SLIT-tablet has comparable efficacy to HDM SCIT. A societal perspective was adopted in the model, including relevant costs of medications, health care services and productivity loss. A 3 year time horizon was used corresponding to a recommended treatment course of allergy immunotherapy. Resource use and costs were based on published sources, where possible, and validated and complemented by a Canadian specialist clinician (allergist) in active practice in Ontario and in Quebec, respectively, where applicable. A discount rate of 1.5% was applied in accordance with the Canadian Agency for Drugs and Technologies in Health (CADTH) guidelines. To assess the robustness of the results, sensitivity analyses were performed by testing alternative assumptions for selected parameters, to understand their impact on the results of the analysis.

**Results:**

The direct treatment costs for a 3-year treatment with SQ HDM SLIT-tablets were higher than for HDM SCIT for both provinces, Ontario and Quebec ($4732.12 and $4829.03 vs. $3434.51 and $2987.74). However, when adding the indirect costs to the model, total savings for the treatment with SQ HDM SLIT-tablets of $1833.00 for Ontario and $769.03 for Quebec were observed. Sensitivity analyses with varying HDM SCIT resource use, discount rates, titration and maintenance injections, nurse time, and number of injections per vial resulted in savings of SQ HDM SLIT-tablets over HDM SCIT in all scenarios analysed.

**Conclusions:**

The CMA indicates that SQ HDM SLIT-tablets are a cost-minimizing alternative to HDM SCIT when considered from a societal perspective in Ontario and Quebec.

## Background

Allergic rhinitis (AR) is an allergic immune response that can be caused by inhaled allergens in sensitized individuals. This condition affects at least 20% of the westernized population with a rising trend of increasing prevalence [1]. In Canada, AR is estimated to affect about 20 to 25% of the population, and more than half of these individuals are not well controlled on conventional symptomatic medications [2].

Several treatment measures and options aiming to reduce the symptoms are available, including allergen avoidance, oral and intranasal antihistamines, intranasal corticosteroids, combination intranasal corticosteroid/antihistamine sprays, leukotriene receptor antagonists (LTRAs), and allergy immunotherapy (AIT) [1, 3, 4]. For patients with persistent AR despite the use of pharmacologic therapies and evidence of specific Immunoglobulin (Ig) E antibodies to clinically relevant allergens, AIT may be considered [1, 3–5].

Allergy immunotherapy involves the repeated administration of the relevant AR-inducing allergen(s) in order to desensitize the patient through a gradual reduction in IgE-mediated responses [6]. Traditionally, AIT is administered as subcutaneous immunotherapy (SCIT), which needs to be administered at a clinic or physician’s office and usually follows a course of 3 to 5 years including a build-up/titration phase followed by maintenance injections [1]. Due to the requirement for frequent visits to a clinic or physician’s office, SCIT is considerably time-consuming, which may result in patients discontinuing therapy. An alternative to the subcutaneous route is sublingual immunotherapy (SLIT), which was first accepted by the WHO in 1998 and the use of which has been increasing globally over the past two decades [7, 8]. However, in Canada, treatment with SLIT-tablets is still evolving, with standardized products only becoming available as recently as 2013 [9]. Benefits over the subcutaneous route include the comfort of avoiding injections, suitability for at-home treatment (once the first dose is tolerated under medical supervision), decreased burden of travel and time off work, as well as a favourable safety profile [1, 5]. Although local side effects are common, severe systemic reactions including anaphylactic reactions are rarely reported [8].

House dust mites (HDMs) are one of the most common sources of indoor allergens and with perennial symptoms HDM-induced AR is usually a chronic or persistent condition [1]. HDM SCIT treatment has a longstanding history of availability in Canada, while the SQ HDM SLIT-tablet (ACARIZAX™) was approved by Health Canada in May 2017. The approval was granted as an AIT option for the treatment of moderate to severe HDM-induced AR, with or without conjunctivitis, in adults 18 to 65 years of age confirmed by a positive skin prick test and/or in vitro IgE antibody testing for *Dermatophagoides pteronyssinus* (*D. pteronyssinus*) or *Dermatophagoides farinae* (*D. farinae*), the most common species in Canada [10].

The cost-minimization analysis (CMA) described here was performed to understand the economic implications of introducing SQ HDM SLIT-tablet in Canada, where HDM SCIT is already an available treatment option.

## Methods

### Cost minimization analysis

The CMA was performed to estimate the economic impact of SQ HDM SLIT-tablet (*D. pteronyssinus* and *D. farinae*, 12 SQ-HDM, ALK-Abello, Denmark) compared to other options available in Canada. Data was gathered for Ontario and Quebec. HDM SCIT is the only appropriate comparator for the analysis. Concomitant use of anti-allergic medicines or other conventional medicines targeted to reduce symptoms of AR were assumed to be the same in SLIT-tablet and SCIT patients and were excluded from the analysis. Based on the evidence available it was assumed that the SQ HDM SLIT-tablet has at least the same efficacy as HDM SCIT, and thus a CMA was deemed appropriate. The underlying assumption of therapeutic equivalence could be considered appropriate given the evidence supporting a favourable safety profile for SLIT vs. SCIT [11, 12]. A societal perspective was adopted in the model, including relevant costs of medications, health care services and productivity loss due to time off work. Therefore costs paid by the government, physician and patient were all included in the analysis. The time horizon in the model was 3 years, which corresponds to a recommended treatment course of AIT [1, 7, 13]. A discount rate of 1.5% was applied, which was in accordance with the Canadian Agency for Drugs and Technologies in Health (CADTH) Guidelines for the Economic Evaluation of Health Technologies [14] and which is a common method in health economic modelling. Inputs were sourced from literature and validated by a Canadian specialist clinician (allergist) in active practice in Ontario and in Quebec, respectively.

### Resource use

Three types of resources were considered in the analysis for each comparator: the medications, services of health care professionals and patient resources. Table 1 gives an overview of the resource use of SQ HDM SLIT-tablet and HDM SCIT during a 3 year course of AIT.

**Table 1:** Resource use for SQ HDM SLIT-tablet and HDM SCIT (Ontario and Quebec)

Resource	Ontario						Quebec					
	SQ HDM SLIT-tablet			HDM SCIT			SQ HDM SLIT-tablet			HDM SCIT		
	Year 1	Year 2	Year 3	Year 1	Year 2	Year 3	Year 1	Year 2	Year 3	Year 1	Year 2	Year 3
10 mL vial^a^ (10 inj.)	–	–	–	3.1	1.3	1.3	–	–	–	3.1	1.3	1.3
SQ HDM SLIT-tablet (package with 30 tablets)	12	12	12	–	–	–	12	12	12	–	–	–
Start-up visits	1	–	–	–	–	–	1	–	–	1	–	–
GP (Ontario 20%, Quebec 5%)^a^	0.2	–	–	–	–	–	0.05	–	–	0.05	–	–
Specialist (Ontario 80%, Quebec 95%)^a^	0.8	–	–	–	–	–	0.95	–	–	0.95	–	–
Titration visits (1 week between inj. [15])	–	–	–	24	–	–	–	–	–	24	–	–
GP (Ontario 20%, Quebec 95%)^a^	–	–	–	4.8	–	–	–	–	–	22.8	–	–
Specialist (Ontario 80%, Quebec 5%)^a^	–	–	–	19.2	–	–	–	–	–	1.2	–	–
Maintenance visits (4 weeks between inj. [15])	–	–	–	7	13	13	–	–	–	7	13	13
GP (95%)^a^	–	–	–	6.65	12.35	12.35	–	–	–	6.65	12.35	12.35
Specialist (5%)^a^	–	–	–	0.35	0.65	0.65	–	–	–	0.35	0.65	0.65
Nurse time ([h]; Ontario 0.5 per inj., Quebec 0.75 per inj.)^a^	–	–	–	15.5	6.5	6.5	–	–	–	23.25	9.75	9.75
Nurse time ([h]; 0.75 per start-up, tablet/only Quebec)	–	–	–	–	–	–	0.75	–	–	–	–	–
Follow-up visits	1	1	1	1	1	1	1	1	1	1	1	1
GP (Ontario 50% SLIT, 20% SCIT; Quebec 50%)^a^	0.5	0.5	0.5	0.2	0.2	0.2	0.5	0.5	0.5	0.5	0.5	0.5
Specialist (Ontario 50% SLIT, 80% SCIT; Quebec 50%)^a^	0.5	0.5	0.5	0.8	0.8	0.8	0.5	0.5	0.5	0.5	0.5	0.5
Patient’s time [h]^a^ [15]	3^c^	1.25^c^	1.25^c^	57.57^b^	24.87^b^	24.87^b^	3^c^	1.25^c^	1.25^c^	48.48^b^	20.53^b^	20.53^b^
Patient’s travel distance ([km]; 20 km per visit) [15]	40	20	20	620	260	260	40	20	20	660	280	280

*GP* general practitioner, *HDM SCIT* house dust mite subcutaneous immunotherapy, *inj.* injection, *SQ HDM SLIT-tablet* SQ house dust mite sublingual immunotherapy tablet

^a^Based on physician input, ^b^ Patient’s time include: travel time round trip 40 min [15], wait time 15 min [15], injection time 4 min^a^, post-injection time 30 min [15], physician consultation time 20 min^a^, ^c^ Patient’s time include: Travel time round trip 40 min [15], wait time 15 min [15], physician consultation time 20 min^a^, and for year 1 only a 30 min post-tablet observational time after first tablet intake [28]

For the SQ HDM SLIT-tablet, the recommended dose is one tablet once daily. For HDM SCIT it was assumed that 24 weekly injections would be given for the titration phase, followed by maintenance injections once every 4 weeks. It was also assumed that one 10 mL vial would last for 10 injections based on findings by Blume et al. [15]. The key difference between these treatments is the at-home administration of the SQ HDM SLIT-tablet resulting in reduced use of services of health care professionals as well as lower costs due to a decline in work productivity. In that respect it was assumed that a SQ HDM SLIT-tablet patient would attend one start-up visit and thereafter not require further use of health care resources for administration. For a typical HDM SCIT patient, 24 titration visits in year 1 and maintenance visits once every 4 weeks thereafter were assumed. Annual follow-up visits were assumed for both.

### Resource costs

The costs of the resources are summarised in Table 2. Assumptions for medication costs were obtained from Ontario Public Drug Programs [16], Régie de l’assurance maladie du Québec (RAMQ) [17] and Association Québécoise des pharmaciens propriétaires (AQPP) [18]. Costs for the services of health care professionals were obtained from the Ontario Ministry of Health and Long Term Care [19], Régie de l’assurance maladie du Québec/Direction des services à la clientèle professionnelle/Manuel des médecins spécialistes [20] and Careers in nursing Canada [21], cost estimates for hours of lost work from Statistics of Canada [22], and travel costs per kilometre was based on national rates for the province or territory for travel reimbursement in private vehicles [23]. All costs are presented in Canadian Dollars.

**Table 2:** Resource costs for SQ HDM SLIT-tablet and HDM SCIT (Ontario and Quebec)

Cost category	Cost type		$CAD/unit	
	Ontario	Quebec	Ontario	Quebec
SQ HDM SLIT-tablet	Box of 30 tablets		117.30	
	Dispensing fee/claim [16, 17]		8.93	9.00
HDM SCIT vials	10 mL concentrate (Omega) [18]		107.64	
Physician	Medical specific re-assessment (follow-up visit), specialist consultation, A134/A624/A474 [19]	Visite principale 09127 (cabinet privé) [20]	61.25	88.50
	Partial assessment (pre- or post-injection), specialist consultation, A138/A628/A478 [19]	Visit de controle 09129 (cabinet privé) [20]	38.05	59.50
	Intermediate assessment, GP consultation, A007	–	33.70	–
	Minor assessment, GP consultation with injection, A001	–	21.70	–
	Injection (sole reason for visit), G202 [19]	Cure d’hyposensibilisation inclunt la partipation de professionelle au procédé, le cas échéant, et l’interpretation, une inejection 00100 (cabinet) [20]	4.45	15.00
	Injection (with consultation at same visit), G212 [19]		9.75	
Nurse	Hourly wage [21]		30.00	
Patient	Average hourly wage [22]		25.79	24.66
	Travel expense by private car [23]		0.51	0.50

*GP* general practitioner, *HDM SCIT* house dust mite subcutaneous immunotherapy, *SQ HDM SLIT-tablet* SQ house dust mite sublingual immunotherapy tablet

To calculate the costs and potential savings associated with the use of SQ HDM SLIT-tablet vs. HDM SCIT over the 3-year time horizon, the costs per unit of each resource is multiplied by the amount of resource used per year.

### Sensitivity analyses

To assess the robustness of the results, sensitivity analyses were performed by testing alternative assumptions for selected parameters, to understand their impact on the results of the analysis.

## Results

### Cost of treatment—SQ HDM SLIT-tablet vs. HDM SCIT

The cost of treatment per year and the results of costs and potential savings associated with the use of a 3-year’s treatment with SQ HDM SLIT-tablet vs. HDM SCIT are summarised in Tables 3 and 4, respectively. Costs of SQ HDM SLIT-tablet treatment per year were similar for each of the 3 years and also between Ontario and Quebec (Table 3). For HDM SCIT treatment, costs in the first year were much higher than in year 2 and 3 of treatment (Table 3): in Ontario it was $3783.70 in the first year compared to $1514.38 in the second and third year, and $3166.48 vs. $1331.88 in Quebec, respectively. The direct costs, including the costs for drug, physician and nurse, for a 3-year’s treatment with SQ HDM SLIT-tablets were higher than for HDM SCIT for both provinces, Ontario and Quebec: for SQ HDM SLIT-tablets it was $4732.12 and $4829.03 compared to $3434.51 and $2987.74, respectively, for HDM SCIT (Table 4). For indirect costs, including patient’s travel expenses and lost working hours, it was the contrary with $180.54 and $173.43 for SQ HDM SLIT-tablets and $3311.14 and $2783.75 for HDM SCIT, respectively. Overall, the CMA revealed total cost savings with SQ HDM SLIT-tablets compared to HDM SCIT of $1833.00 for Ontario and $769.03 for Quebec over 3 years of treatment (Table 4).

**Table 3:** Costs of SQ HDM SLIT-tablet and HDM SCIT treatment per year (Ontario and Quebec; in $ CAD)

Cost category	Ontario			Quebec		
	Year 1	Year 2	Year 3	Year 1	Year 2	Year 3
SQ HDM SLIT-tablet						
Drug costs	1534.58	1534.58	1534.58	1536.65	1536.65	1536.65
Tablet costs	1427.15	1427.15	1427.15	1427.15	1427.15	1427.15
Dispensing fee	107.43	107.43	107.43	109.50	109.50	109.50
Physician costs	103.22	47.48	47.48	148.00	59.50	59.50
GP costs	23.59	16.85	16.85	34.18	29.75	29.75
Specialists costs	79.63	30.63	30.63	113.83	29.75	29.75
Nurse costs	–	–	–	22.50	0.00	0.00
Total health care costs	1637.80	1582.06	1582.06	1707.15	1596.15	1596.15
Patients costs	97.65	42.38	42.38	93.78	40.73	40.73
Time costs	77.37	32.24	32.24	73.98	30.83	30.83
Travel costs	20.28	10.14	10.14	19.80	9.90	9.90
Total costs	1735.45	1624.43	1624.43	1800.93	1636.88	1636.88
HDM SCIT						
Drug costs	333.68	139.93	139.93	333.68	139.93	139.93
Physician costs	1186.03	406.32	406.32	613.00	254.50	254.50
Injection with consultation costs (Ontario), injection only (Quebec)	1130.29	350.58	350.58	465.00	195.00	195.00
GP costs	299.42	322.95	322.95	441.75	185.25	185.25
Specialists costs	830.88	27.63	27.63	23.25	9.75	9.75
Consultation only costs	55.74	55.74	55.74	148.00	59.50	59.50
GP costs	6.74	6.74	6.74	34.18	29.75	29.75
Specialists costs	49.00	49.00	49.00	113.83	29.75	29.75
Nurse costs	465.00	195.00	195.00	697.50	292.50	292.50
Total Health care costs	1984.72	741.25	741.25	1644.18	686.93	686.93
Patients costs	1798.98	773.13	773.13	1522.30	644.95	644.95
Time costs	1484.64	641.31	641.31	1195.60	506.35	506.35
Travel costs	314.34	131.82	131.82	326.70	138.60	138.60
Total costs	3783.70	1514.38	1514.38	3166.48	1331.88	1331.88

*GP* general practitioner, *HDM SCIT* house dust mite subcutaneous immunotherapy, *SQ HDM SLIT-tablet* SQ house dust mite sublingual immunotherapy tablet

**Table 4:** Costs and potential savings of 3 year’s treatment: SQ HDM SLIT-tablet vs. HDM SCIT (Ontario and Quebec; in $CAD)

Cost category	Ontario			Quebec		
	SQ HDM SLIT-tablet	HDM SCIT	SQ HDM SLIT-tablet vs. HDM SCIT	SQ HDM SLIT-tablet	HDM SCIT	SQ HDM SLIT-tablet vs. HDM SCIT
Drug costs	4536.04	607.37	3928.67	4542.16	607.37	3934.78
Physician costs	196.07	1980.74	− 1784.67	264.38	1110.77	− 846.40
Nurse costs	–	846.40	− 846.39	22.50	1269.60	− 1247.09
Total health care costs	4732.12	3434.51	1297.60	4829.03	2987.74	1841.29
Indirect costs (patient)	180.54	3311.14	− 3130.60	173.43	2783.75	− 2610.32
Total costs	4912.65	6745.65	− 1833.00	5002.47	5771.49	− 769.03

*HDM SCIT* house dust mite subcutaneous immunotherapy, *SQ HDM SLIT-tablet* SQ house dust mite sublingual immunotherapy tablet

### Sensitivity analyses

**Fig. 1:**
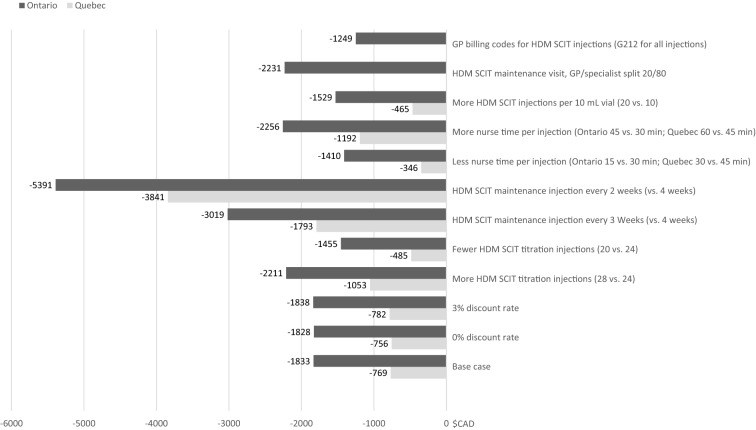
Sensitivity analyses: potential total savings of SQ HDM SLIT-tablet vs. HDM SCIT (Ontario and Quebec). Potential total savings according to total costs. Total cost of 3-year’s treatment are discounted with 1.5% as described in methods, except for the sensitivity analyses “0% discount rate” and “3% discount rate” where the parameter discount rate was changed to 0% or 3%, respectively. GP general practitioner, HDM SCIT house dust mite subcutaneous immunotherapy

Results of the sensitivity analyses are shown in Fig. 1. Overall, the sensitivity analyses demonstrated cost savings with SQ HDM SLIT-tablets compared to HDM SCIT, with some variation in the magnitude of potential savings. Results for the cost difference for a 3 year treatment of SQ HDM SLIT-tablets compared to HDM SCIT was sensitive to changes in nurse time per injection, number of HDM SCIT titration injections, and number of injections per vial. When the nurse time was reduced from 30 to 15 min (Ontario) or 45 to 30 min (Quebec), the potential savings with SQ HDM SLIT-tablets vs. HDM SCIT were $1409.80 and $345.83, respectively. If fewer HDM SCIT titration injections were used (20 vs. 24 injections), the potential savings were $1454.78 (Ontario) and $484.80 (Quebec). If more HDM SCIT injections per 10 mL vial were used (20 vs. 10 injections), the potential savings were $1529.32 (Ontario) and $465.34 (Quebec). However, for Ontario, the most conservative scenario where the G212 billing code (injection as sole reason for visit) was charged by general practitioners for all injections resulted in savings of $1249.08, corresponding to savings of $1860.47 for the first year and a subsequent over cost of $312.59 both for second and third year (due to different practices in how these visits are billed by physicians, this was analysed only for Ontario). By far the biggest cost difference between SQ HDM SLIT-tablet and HDM SCIT treatment was observed for the scenario of HDM SCIT maintenance injections every 2 weeks instead of every 4 weeks with potential savings of $5390.94 (Ontario) and $3841.21 (Quebec), respectively. As expected, almost no impact to the cost difference compared to the base case was observed if the discount rate was changed (from 1.5 to either 0% or 3%) in the sensitivity analyses as most costs are seen in the first year of treatment.

## Discussion

The current CMA analysed the economic impact of SQ HDM SLIT-tablet compared to HDM SCIT in Canada for the treatment of HDM-induced AR. Overall, the presented results indicate a cost-minimizing potential of SQ HDM SLIT-tablets for the treatment of HDM-induced AR when compared to HDM SCIT. Comparing only the direct costs, treatment with SQ HDM SLIT-tablets were higher than that for HDM SCIT, which is driven by the higher drug costs for SQ HDM SLIT-tablets. In addition, a cost difference was also observed between Ontario and Quebec: the costs for SQ HDM SLIT-tablet treatment were comparable between Ontario and Quebec, but savings in direct costs when using HDM SCIT treatment were less for Ontario. Even though the costs associated with nurse time were lower in Ontario due to the assumptions made by the Canadian specialist clinicians for each province (30 min vs. 45 min nurse time), the observed cost difference is due to the physician costs for HDM SCIT treatment in Ontario, which were almost twice as high as for Quebec. Although according to the province guidelines and manuals the physician costs per unit are higher in Quebec, in Ontario physicians charge an injection fee in addition to a consultation fee. In contrast, in Quebec only the injection fee can be charged, even if the visit includes a consultation. The impact of the physician costs is also apparent looking at the most conservative scenario for Ontario where the G212 billing code is charged by physicians for all injections resulting in the least cost difference between the treatment with SQ HDM SLIT-tablets or HDM SCIT. Nevertheless, cost savings are still observed in this scenario.

Adding the indirect costs to the analysis, which are much higher for HDM SCIT than SQ HDM SLIT-tablet due to the frequent visits to a clinic or physician’s office, resulted in overall potential savings of $2408.41 for Ontario and $769.03 for Quebec. Therefore, looking at the total costs the CMA indicates that treatment with SQ HDM SLIT-tablet is a cost-minimizing alternative to HDM SCIT in Canada. This is in line with recent reports, which state that in four of six studies comparing cost outcomes of SLIT vs. SCIT, SLIT was the cost-saving therapy [24].

As HDM SCIT treatment requires a much higher number of clinic visits, this subsequently results in both higher costs associated with health care professional services and patient resources, thus outweighing the higher drug costs for the SQ HDM SLIT-tablet. Looking at the annual costs, most of the resource use differences for HDM SCIT treatment were observed in the first year of treatment, which is attributed to the 24 weekly titration visits. With the difference in the number of clinic visits between SQ HDM SLIT-tablet and HDM SCIT treatment being the main driver of the cost difference, parameters affecting the treatment setting had a relatively large impact in the sensitivity analyses. In addition, at-home administration of SQ HDM SLIT-tablet is more convenient for patients as it decreases the burden of travel and time off work. The latter also being an advantage in reducing the economic burden to society as the target population for the treatment mainly belongs to the working population. At-home administration of SLIT-tablet can be particularly advantageous in rural communities, where large distances from the nearest clinic may pose additional barriers to access and, with a high number of visits required for SCIT, potentially reducing the likeliness of patients to continue this therapy. A patient preference study conducted in Germany using a discrete choice experiment in 239 adults with moderate to severe grass, birch, and/or HDM AR found that the attribute most preferred by patients regarding the mode of AIT administration was the number and duration of physician visits, with a strong preference for fewer visits with shorter duration [25].

Practice guidelines recommend a standardized dosing approach for SCIT flexibility to individual needs as acknowledged [6, 9, 13]. This flexibility was recently demonstrated in US and Canadian practice where a wide variety in SCIT treatment regimens for AR was being used [15]. Therefore, depending on the specific patient’s needs and also the preferences for titration dosing schedule of the administering physician, it is possible that some patients would receive slightly more or fewer titration doses upon treatment initiation. In addition, the patient’s maintenance dosing schedule may vary, with product labels recommending a 2 to 4-week maintenance dose regimen. Due to the costs associated with each injection visit, varying both the number of titration injections and frequency of maintenance injection had an important impact on the results of the sensitivity analyses. Indeed, the scenario of HDM SCIT maintenance injections every 2 weeks instead of every 4 weeks resulted in the biggest potential savings ($5390.94 for Ontario and $3841.21 for Quebec) for SQ HDM SLIT-tablet vs. HDM SCIT treatment. However, from clinical experience, it has to be noted that probably only a minority of 10 to 15% would follow a treatment schedule with maintenance injections every 2 weeks. Overall, the cost of treatment of SQ HDM SLIT-tablet was lower than for HDM SCIT for each of the scenarios analysed.

As SLIT-tablets are a relatively novel approach to AIT in Canada [9], SCIT is more commonly used. In North America, as opposed to Europe, it is more common to combine more than one allergen into the same SCIT injection [15]. Important to note in this context is, that efficacy of the SQ HDM SLIT-tablet in a North American field trial was shown to be effective and well tolerated in subjects with HDM allergy, most of whom (76%) were polysensitized to allergens other than HDM, and efficacy in HDM-monosensitized and polysensitized subjects was comparable [26]. Similarly, this has also been shown in European field trials [11, 27]. SQ HDM SLIT-tablets are a safe, effective, and convenient option for patients with HDM AR. Generally, the SQ HDM SLIT-tablet represents an improved AIT treatment option providing some advantages over HDM SCIT, including the need for fewer clinic visits, a better safety profile and a standardized quality allergen formulation, with the potential to increase access to AIT for the treatment of HDM-induced AR.

This study has some limitations. Cost and resource use included in the analysis were solely associated to the treatment itself and administration of the treatment, including direct and indirect costs. However, as the intent was to analyse the economic impact of SQ HDM SLIT-tablet compared to HDM SCIT in Canada, it does not depict other aspects of HDM-induced AR. In addition, certain assumptions made for the resource use were based on Canadian specialist clinician input for each, Ontario and Quebec. Finally, a CMA builds on an assumption of equal efficacy. Given the lack of head to head studies, this is a recognized limitation of this analysis, however this issue is not uncommon when products are still relatively new in the market.

## Conclusions

The CMA to estimate the economic impact of SQ HDM SLIT-tablet compared to other options available (HDM SCIT) in Canada for the treatment of HDM-induced AR indicates that SQ HDM SLIT-tablets are a cost-minimizing alternative to HDM SCIT when considered form a societal perspective in both analysed provinces, Ontario and Quebec. Sensitivity analyses with varying HDM SCIT resource use, discount rates, titration and maintenance injections, nurse time, and number of injections per vial support this conclusion, where all analysed scenarios still resulted in savings of SQ HDM SLIT-tablets over HDM SCIT. The CMA demonstrates the economic impact introducing SQ HDM SLIT-tablets in Canada, however, further analyses including other aspects of HDM-induced AR may be needed.

## Abbreviations

AIT: allergy immunotherapy
AQPP: Association Québécoise des pharmaciens propriétaires
AR: allergic rhinitis
CADTH: Canadian Agency for Drugs and Technologies in Health
CMA: cost minimization analysis
GP: general practitioner
HDM: house dust mite
Ig: immunoglobulin
LTRA: leukotriene receptor antagonist
RAMQ: Régie de l’assurance maladie du Québec
SCIT: subcutaneous immunotherapy
SLIT: sublingual immunotherapy
